# Allograft inflammatory factor 1 (AIF-1) is a new human adipokine involved in adipose inflammation in obese women

**DOI:** 10.1186/1472-6823-13-54

**Published:** 2013-11-25

**Authors:** Silvia Lorente-Cebrián, Pauline Decaunes, Elisabeth Dungner, Anne Bouloumié, Peter Arner, Ingrid Dahlman

**Affiliations:** 1 Department of Medicine Huddinge, Lipid Laboratory, Novum, Karolinska Institutet, Karolinska University Hospital, SE-141 86 Huddinge, Stockholm, Sweden; 2 Institut National de la Santé et de la Recherche Médicale (INSERM), U1048, Institut des maladies cardiovasculaires et métaboliques, Université Toulouse III Paul-Sabatier, Toulouse, France

**Keywords:** AIF-1, Adipokines, Obesity, Adipose tissue

## Abstract

**Background:**

Allograft inflammatory factor 1 (AIF-1) is a putative obesity gene. Our aim was to examine the expression of AIF-1 in human white adipose tissue (WAT) in relation to obesity and metabolic phenotypes in women.

**Methods:**

WAT secretion of AIF-1 was determined in subcutaneous adipose tissue pieces *in vitro* by ELISA from 5 subjects. mRNA expression of AIF-1 was determined by RT-qPCR in the isolated cell fractions of adipose tissue (n = 5-6 *per* group), in subcutaneous and visceral WAT pieces from non-obese (n = 12) and obese women (n = 23), and in some subcutaneous WAT also before and after weight reduction (n = 10). Finally, adipose AIF-1 mRNA was related to metabolic phenotypes in 96 subjects with a wide range of BMI.

**Results:**

AIF-1 was secreted in a time dependent fashion from WAT. The major source of AIF-1 was WAT resident macrophages. Expression of AIF-1 was similar in visceral and subcutaneous WAT and was two-fold increased in obese women (P < 0.01). AIF-1 mRNA expression levels were normalized after weight reduction (P < 0.01). Expression of AIF-1 was inversely correlated with insulin sensitivity as assessed by insulin tolerance test (KITT), and circulating levels of adiponectin (P = 0.02), and positively correlated with insulin resistance as estimated by HOMA (=0.0042).

**Conclusions:**

AIF-1 is a novel adipokine produced mainly by macrophages within human WAT. Its expression is increased in obese women and associates with unfavourable metabolic phenotypes. AIF-1 may play a paracrine role in the regulation of WAT function through cross-talk between macrophages and other cell types within the adipose tissue.

## Background

In obese subjects, dysfunctional adipose tissue is believed to contribute to metabolic disturbances including insulin resistance. “Harmful” adipose tissue is characterized by hypertrophic large fat cells, increased lipolysis, low grade inflammation, and altered release of adipose hormons i.e. adipokines [1-4]. Most adipokines display increased release in obesity but some, such as adiponectin which has beneficial metabolic effects in rodent models, are decreased in obese subjects [5].

Human adipose tissue is composed of several cell types like adipocytes, endothelial and progenitor cells, and monocytes/macrophages that interact to regulate adipose tissue function [6]. Obesity is associated with infiltration of activated macrophages in adipose tissue, which besides contributing to insulin resistance [4,7] may also be directly involved in regulation of fat mass [8]. Adipose tissue macrophages produce a number of pro-inflammatory cytokines that promote adipose dysfunction and insulin-resistance [4,7]. To date, a number of cytokines have been described to be secreted from different cells in human adipose tissue [9,10].

Allograft inflammatory factor-1 (AIF-1) is a protein which participates in inflammatory responses and regulates the function of macrophages [11]. Recent studies have demonstrated that AIF-1 can stimulate the release of inflammatory cytokines in mouse macrophages [12] although its mechanism of action is incompletely understood. AIF-1 has both been reported to be an intra-cellular calcium-binding protein [13] and to be a secreted factor [14,15], which may be linked to the presence of different splice variants of AIF-1 gene in humans [11,12].

Recent findings suggest a role for AIF-1 in obesity. First, a single nucleotide polymorphism (SNP) in the AIF-1 gene region has been associated with body weight in a large genome wide-association study [16]. Second, AIF-1^−/−^ mice are protected against diet induced obesity [17]. Based on these findings, in this project we aim to explore the potential function of AIF-1 in female adipose tissue by mapping AIF-1 expression and possible release from cells within adipose tissue, as well as its relationship to obesity and metabolic disturbances.

## Methods

### Participants and clinical evaluation

Subjects were either recruited by local advertisement or from the outpatient clinic for treatment of obesity at the local hospital. Obesity was defined as body mass index (BMI) 30 kg/m^2^ or greater. All subjects were healthy except for obesity and were free of medication. Participants were investigated in the morning after an overnight fast. The study was approved by the Research Ethics Committee at Karolinska Institutet, South (Stockholm) and the Institutional Research Board of INSERM and Toulouse University Hospital. Written informed consent was obtained from all participants. All the experimental procedures were conducted under the provisions of the Declaration of Helsinki.

Examined cohorts are described in Table 1. All subjects comprised women. Cohort 1 comprised five women (age 44 ± 3 yr, BMI 33 ± 11 kg/m^2^) that was used to evaluate *in vitro* secretion of AIF-1 from abdominal adipose pieces. WAT was obtained in connection with surgery for cosmetic reasons or gastric bypass surgery for treating obesity. These samples had been previously collected.

**Table 1:** Description of the cohorts

**Cohort**	**Gender (F/M)**	**Age (years)**	**BMI (kg/m**^**2**^**)**	**fP-glucose (mmol/L)**	**fS-insulin (μg/ml)**	**KITT (%/min)**	**S-adiponectin (μg/ml)**	**T-cholesterol (mmol/L)**
1	5/0	44 ± 3	33 ± 11	N.A.	N.A.	N.A.	N.A.	N.A.
2^1^	28/0	41 ± 2	29 ± 1	N.A.	N.A.	N.A.	N.A.	N.A.
3 (obese)	23/0	42 ± 10	44 ± 4	5.80 + 1.00	20.08 + 11.62	2.95 + 0.78	N.A.	5.13 + 0.75
3 (non-obese)	12/0	40 ± 13	24 ± 2	4.82 + 0.42	8.45 + 2.91	N.A.	N.A.	4.91 + 0.99
4	10/0	39 ± 6	40 ± 6	5.61 + 0.67	15.76 + 6.29	3.12 + 0.40	N.A.	4.83 + 0.75
5	96/0	39 ± 9	38 ± 7	5.51 + 1.06	14.50 + 9.09	3.54 + 1.37^2^	12.04 + 5.85^3^	5.00 + 0.95

^1^From each subject only one cell type could be isolated from adipose tissue. Five to six subjects were used to quantify each of the different sub-fraction of cells in adipose tissue. In total, 28 subjects were used. ^2^KITT was available for 46 women. ^3^S-Adiponectin was available for 37 women.

N. A. = Not available.

The cellular origin of AIF-1 was measured in cohort 2 comprising women undergoing abdominal dermolipectomy for cosmetic reasons (age 41 ± 2 yr, BMI 29 ± 1 kg/m^2^). Additional clinical data was not available for this cohort. From each subject only one cell type could be isolated from adipose tissue. Five to six subjects were used to quantify each of the different sub-fractions of cells in adipose tissue. In total, 28 subjects were used.

AIF-1 mRNA levels were quantified in subcutaneous and visceral WAT in cohort 3 comprising 12 non-obese (age 40 ± 13 yr, BMI 24 ± 2 kg/m^2^) and 23 obese women (age 42 ± 10 yr, BMI 44 ± 4 kg/m^2^). The non-obese subjects were operated for uncomplicated gallstone disease and the obese underwent anti-obesity (bariatric) surgery (BMI > 40). Subcutaneous adipose tissue from the surgical incision and omental (visceral) adipose tissue were obtained at the beginning of surgery. Only saline was given as an intravenous infusion until adipose tissue was removed.

Subcutaneous WAT AIF-1 mRNA levels were further investigated in cohort 4 comprising 10 obese women (aged 39 ± 6 yr, BMI 40 ± 6 kg/m^2^) investigated before and 2–4 yr after intense anti-obesity therapy with anti-obesity surgery or behavioral modification when they had reached a non-obese weight-stable state for at least 6 months (BMI 26 ± 3 kg/m^2^) [18]. Briefly, adjustable vertical gastric banding was used as surgical treatment. The patients were followed yearly and re-examined when body weight had reached nadir two to four years after operation and was stabilized for at least 6 months according to self report (< 1 kg increase or decrease). Behavioral modification consisted of increasing motivation, changing eating habits, enhancing physical activity and regular 3 month follow ups for a year, when re-examination took place. By then body was stabilized for at least one month according to self report (< 1 kg increase or decrease). Weight loss was 22 ± 12% (mean ± SD) in the surgery group and 14 ± 6% in the behavioural group. AIF-1 mRNA levels were related to metabolic phenotypes in cohort 5 comprising n = 96 women with a wide variation in BMI (age 39 ± 9 yr, BMI 38 ± 7 kg/m^2^). In cohorts 4 and 5 biopsies of the subcutaneous abdominal WAT (0.5–2 g) were obtained by needle aspiration under local anesthesia. Cohorts 1, 3, 4 and 5 comprised third-generation Caucasian Swedish women living in Stockholm. Cohort 2 comprised Caucasian French women. WAT samples were brought to the laboratory in saline, and one part was used immediately for adipocyte experiments and another part was frozen in liquid nitrogen. Unfortunately, it was not possible to recruit enough male subjects to perform these clinical studies.

### Insulin tolerance test (KITT) and HOMA_IR_

Insulin tolerance test was performed as previously described [19]. Briefly, patients were examined in the hospital at 8 a.m. after an overnight fast. Following a 30–60 min rest, venous blood samples were taken for determinations of fasting levels of glucose and plasma insulin. Next, insulin was rapidly injected intravenously. After 2.5, 5, 7.5, 10, 15, 20, 25 and 30 min’s, blood glucose levels were determined and plotted in a semilogaritmic graph depicting blood glucose decrease along time. The rate constant (k) derived from this plot represented the intravenous insulin tolerance (KITT). The homeostasis model assessment for insulin resistance (HOMA_IR_) was calculated as fasting serum insulin (μU/ml) x fasting plasma glucose (mmol/L)/22.5 [20].

### Adipose morphology

Morphology of adipose tissue was calculated as previously described [21]. Briefly, adipocytes and stroma-vascular fraction (SVF), respectively, were isolated from adipose tissue by collagenase digestion as previously described and mean fat cell size determined [22]. Total fat mass was measured by bioimpedance. We have fitted a curve, which defines the relationship between mean adipocyte volume and fat mass. The differences between observed mean adipocyte volume in the present investigation and the expected volume as obtained from the curve fit at the corresponding level of total body fat mass were calculated separately for each of the 96 woman. The women were classified as having either hyperplastic (negative deviation) or hypertrophic (positive deviation) adipose tissue morphology relative to the estimated average value for body fat. The morphology values are quantitative: a larger absolute positive or negative value reflects more pronounced hypertrophy or hyperplasia, respectively, compared with a small value.

### Isolation of the different cell types from adipose tissue

The stroma-vascular fraction and adipocytes were obtained after collagenase digestion and the different cell types of the stroma-vascular fraction were obtained by immunoselection and depletion as described previously [23,24]. The following cell types were identified: endothelial cells (CD34+/CD31+), fraction containing cells with capacity to differentiate into fat cells (progenitor cells; CD34+/CD31-), macrophages (CD34-/CD14+), and T lymphocytes (CD34-/CD14-/CD31+) [6].

### RNA preparation and cDNA synthesis

Adipose tissue pieces (300 mg) or 200 μL isolated cells were kept at −70°C for subsequent RNA extraction using the RNeasy minikit (QIAGEN, Hilden, Germany). RNA samples were treated with ribonuclease-free deoxyribonuclease (QIAGEN). The RNA concentration was determined using spectrophotometer and the quality of RNA was confirmed using an Agilent 2100 Bioanalyzer (Agilent Technologies, Palo Alto, CA). One microgram of RNA was reverse transcribed to cDNA using the Omniscript reverse transcriptase kit (QIAGEN) and random hexamer primers. For qPCR analysis on the distinct cell types of human adipose tissue, mRNA were extracted from macrophages, lymphocytes, adipocytes, endothelial cells, and progenitor cells isolated from human AT as previously described [6]. RNA was reverse-transcribed using the “Superscript II” kit (Invitrogen, Cergy-Pontoise, France).

### Quantitative real-time PCR

Adipose tissue AIF-1 mRNA and the internal control gene 18S were quantified using the SYBR Green-based technology. Primer pairs were designed to span exon-intron boundaries. The following primers were used for specific mRNA quantification: 5’ GATGATGCTGGGCAAGAGAT-3’ and 5’ CCTTCAAATCAGGGCAACTC-3’ for AIF-1; 5’ CACATGGCCTCCAAGGAGTAAG-3’ and 5’ CCAGCAGTGAGGGTCTCTCT-3’ for 18S [25]. 5 ng of cDNA were mixed with gene-specific primers (final concentration 300 nM) and IQ SYBR green supermix (Bio-Rad Laboratories, Hercules, CA) PCR was performed with an iCycler IQ (Bio-Rad Laboratories). A direct comparative method was used for data analysis, *i.e.* 2^(Ct target calibrator - Ct target sample)^/2^(Ct 18S calibrator - Ct 18S sample)^. The PCR efficiency in all runs was close to 100% and all samples were run in duplicate.

For qPCR analysis on the distinct cell types of human adipose tissue AIF-1 primers were from Applied Biosystems (Courtaboeuf, France Hs 00610419 g1). The amplification reaction was performed in duplicate on 15 ng cDNA samples in a final volume of 20 μL in 96-well reaction plates (Applied Biosystems) in a GeneAmp 7500 detection system. All reactions were carried out under the same conditions: 50°C for 2 min, 95°C for 10 min, 40 cycles of 95°C for 15 sec and 60°C for 1 min. Results were analyzed with the GeneAmp 7500 software and all the values were normalized to the levels of 18S rRNA (Applied Biosystems).

### Protein measurements

WAT pieces (300 mg in 3 mL medium) were incubated for different periods of time as described [26]. Briefly, adipose tissue samples from cohort 1 were minced in small pieces and visible vessels and coagulation particles were removed. The remaining tissue (~300 mg) was rinsed in saline at 37°C and incubated in 3 mL Krebs-Ringer phosphate buffer (pH 7.4) supplemented with 40 g/liter of defatted bovine serum albumin and 1 g/liter of glucose. WAT samples were incubated for 3 hours at 37°C in a shaking water bath, with air as the gas phase. After 1, 2 and 3 hours, 2 mL of the medium was removed and frozen in liquid nitrogen. Medium was saved at −70°C for later determination of AIF-1 levels by ELISA (Catalog no. E92288Hu; USCNK Life Science Inc., China), according to the manufacturer’s instructions. Values were expressed as picograms *per* milliliter. Serum levels of adiponectin were measured using a RIA method (Linco Research, Inc., St. Charles, MO) and were expressed as micrograms *per* milliliter.

### Statistical analysis

Unpaired or paired *t* test (two sided) was used to compare mRNA levels between two groups. Values are mean ± SD. We used simple regression and multiple regression with BMI as covariate to examine relationships between quantitative variables. Differences were considered as statistically significant at p < 0.05. The statistical analyses were performed using Statview software (version 5.01; SAS Institute, Cary, NC).

## Results

### AIF-1 is secreted from pieces of adipose tissue

**Figure 1:**
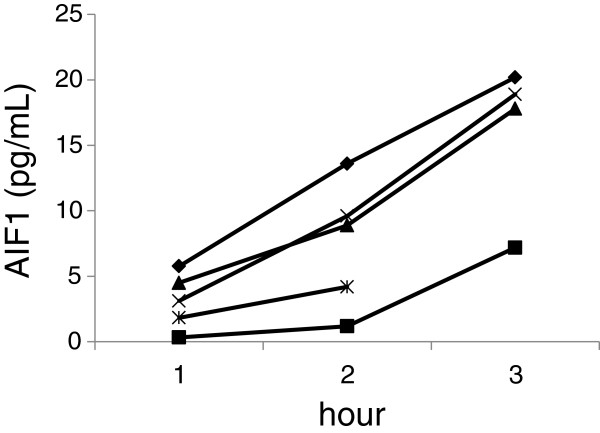
**AIF-1 secretion from WAT *in vitro*.** Pieces of WAT (300 mg) were incubated in Krebs-Ringer phosphate buffer (pH 7.4) supplemented with 40 g/liter of defatted bovine serum albumin and 1 g/liter of glucose (3 mL) and the concentration of AIF-1 in medium was determined by ELISA, see “Protein measurements” section for details (cohort 1; n = 5 women, 44 ± 3 years, BMI 33 ± 11 kg/m^2^). One sample at the 3 h incubation time point was lost during sample processing.

We first wanted to assess whether AIF-1 was secreted from human adipose tissue. Adipose tissue pieces from women in cohort 1 were incubated *in vitro* as described [27] and secretion of AIF-1 was measured in the conditioned medium by ELISA. AIF-1 levels accumulated in the media along the 3 h-experimental period (Figure 1) demonstrating that AIF-1 was secreted in a time dependent-manner from WAT pieces in all of the 5 experiments. This defines AIF-1 as a novel adipokine.

### AIF-1 is mainly produced by adipose-tissue macrophages

**Figure 2:**
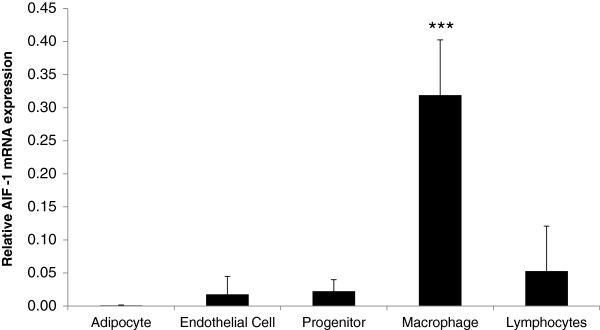
**AIF-1 mRNA levels in the different cell fractions in human WAT (cohort 2; n = 5-6 subjects *per *cell fraction, n = 28 subjects in total, see “Participants and clinical evaluation” section for details).** Statistical differences were analyzed by unpaired t-test and related to the adipocyte fraction. ***P < 0.001 *vs*. adipocyte fraction.

We next investigated the cellular source of AIF-1 in human WAT using cohort 2 (age 41 ± 2 yr, BMI 29 ± 1 kg/m^2^). Our results show that the stroma-vascular fraction was the major source of AIF-1 mRNA in WAT. Highest levels of AIF-1 were observed in the CD34-/CD14+ cells, which contain macrophages (*P* < 0.001; Figure 2). Very low levels of AIF-1 mRNA were detected in isolated adipocytes; mRNA levels in lymphocytes, endothelial and progenitor cells were in between those in adipocytes and macrophages.

### AIF-1 mRNA expression in human adipose tissue in relationship to obesity

**Figure 3:**
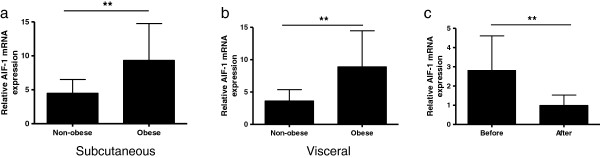
**AIF-1 mRNA levels in relation to adiposity in (a) abdominal subcutaneous WAT, and (b) visceral WAT (cohort 3, n = 23 obese women, 42 ± 10 years, 44 ± 4 kg/m**^**2**^**, n = 12 non-obese women, 40 ± 13 years, 24 ± 2 kg/m**^**2**^**), and (c) in abdominal subcutaneous WAT of obese women (cohort 4, n = 10, 39 ± 6 years, 40 ± 6 kg/m**^**2**^**) before and 2–4 years after intense anti-obesity therapy when they had reached a non-obese weight-stable state.** Statistical differences were analyzed by un-paired **(a and b)** or paired t-test **(c)**. **P < 0.01.

WAT mRNA levels of AIF-1 were increased about two-fold in obese as compared to non-obese women from cohort 3 in both abdominal subcutaneous and visceral fat depots, (*P* < 0.01; Figure 3a and 3b). Neither age, nor adipose tissue depot influenced AIF-1 mRNA expression (values not shown). Furthermore, AIF-1 mRNA levels in subcutaneous WAT were normalized in obese women from cohort 4 following marked long term weight loss (*P <* 0.01; Figure 3c). To assess whether the association between AIF-1 mRNA levels and obesity was related to increased macrophage infiltration into adipose tissue, we wished to correlate AIF-1 levels to a macrophage marker. Integrin alpha-X (ITGAX) is a gene specific for proinflammatory macrophages [28]. We used a previously published global microarray-based gene expression profile on abdominal subcutaneous adipose tissue from obese and lean women (n = 56) [28] to show that AIF-1 and ITGAX levels were strongly correlated (r^2^ = 0.523, P < 0.0001).

### Adipose AIF-1 mRNA expression is correlated with unfavourable clinical metabolic parameters

**Figure 4:**
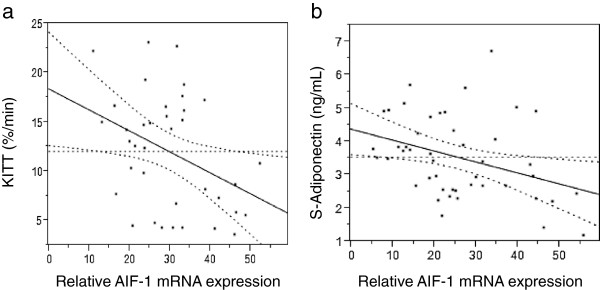
**Correlation between AIF-1 mRNA expression and (a) rate constant of KITT (a subset of cohort 5, n = 46 women, 39 ± 9 years, 39 ± 7 kg/m**^**2**^**) and (b) S-Adiponectin (subset of cohort 5, n = 37 women , 40 ± 8 years, 37 ± 7 kg/m**^**2**^**).** Statistical outcomes are displayed in Table 2.

We next wanted to investigate whether AIF-1 expression in WAT was associated with clinical parameters related to insulin sensitivity in a large cohort of women (cohort 5). AIF-1 was correlated to BMI and not age in this cohort (Table 2). In multiple regression with BMI and AIF-1 mRNA levels as independent factors, we found that WAT AIF-1 expression was independently correlated with insulin resistance as determined by HOMA_IR_ (partial r = 0.369, P = 0.0042) in this cohort of women. In a subgroup of women insulin sensitivity as estimated by the short insulin tolerance test (KITT) and circulating levels of adiponectin had been quantified. In this group of patients WAT AIF-1 expression was inversely and independently of BMI (Table 2) correlated with insulin sensitivity as estimated by the short insulin tolerance test (KITT) (partial r = −0.396, P = 0.017, Figure 4a), and circulating levels of adiponectin (partial r = −0.334, P = 0.023, Figure 4b). Finally, WAT AIF-1 expression was independently of BMI associated with fat cell volume (partial r = 0.396, P = 0.0041; Table 2), but not adipose morphology (values not shown). In the multiple regression analysis (Table 2), BMI became non-significant as regressor in the presence of all variables except adiponectin.

**Table 2:** Regression of clinical and metabolic phenotypes on AIF-1 expression

	**AIF-1**^**1**^		**AIF-1**^**2**^	
**Regressor**	**r -value**	**p value**	**Partial r-value**	**p value**
**Age (years, n = 96)**	0.056	0.59	-	-
**BMI (kg/m**^**2**^**, n = 96)**	0.44	<0.001	-	-
**KITT (rate constants, n = 46)**^**3**^	−0.378	0.0096	−0.396	0.017
**Adiponectin (μg/ml, n = 37)**^**4**^	−0.375	0.022	−0.334	0.023
**Adipose morphology (delta, n = 96)**^**5**^	0.274	0.0068	0.148	0.13
**Fat cell volume (n = 96)**^**6**^	0.456	<0.0001	0.396	0.0041
**Log HOMA (n = 93)**^**7**^	0.497	<0.0001	0.369	0.0042

^1^Simple regression. ^2^Multiple regression with BMI together with one other variable at a time as independent variables. In the multiple regression, BMI became non-significant when examined together with KITT, fat cell volume or HOMA (partial r between 0.04 and 0.18). However it remained significant together with adiponectin (partial r = 0.44; p = 0.004) ^3^Insulin tolerance test was performed in patients after an overnight fast. Insulin was rapidly injected intravenously. After 2.5, 5, 7.5, 10, 15, 20, 25 and 30 min’s, blood glucose levels were determined and plotted in a semilogaritmic graph depicting blood glucose decrease along time. The rate constant (k) derived from this plot represented the intravenous insulin tolerance (KITT). ^4^Serum levels of adiponectin were measured using a RIA method. ^5^The relationship between mean adipocyte volume and total body fat determines adipocyte morphology. Delta is the difference between observed adipocyte volume in the present investigation and the expected volume (as obtained from the curve fit) at the corresponding level of total body fat mass were calculated separately for each woman. The morphology values are quantitative: a larger absolute positive or negative value (delta) reflects more pronounced hypertrophy or hyperplasia, respectively, compared with a small value, see Morphology in methods section for details. ^6^Adipocytes were isolated from adipose tissue by collagenase digestion as previously described and mean fat cell size determined as described in reference [21]. ^7^The homeostasis model assessment for insulin resistance (HOMA_IR_) was calculated as fasting serum insulin (μU/ml) x fasting plasma glucose (mmol/L)/22.5.

## Discussion

Recent proteomics studies suggested that fat cells or other cells within WAT have the potential to generate hundreds of adipokines [10,29,30]. However, only a few of these candidates have been validated as being true adipokines (time dependent secretion) and the regulation or function is unknown for most of the potential adipokines [10]. In the present study, we present evidence that AIF-1 is released from WAT explants in a time-dependent manner indicating that AIF-1 is an adipokine in humans*.* It has previously been shown that the protein expression of AIF1 reflects mRNA levels [31]. In subsequent studies investigating clinical correlates of adipose AIF-1 we therefore relied on mRNA measurements. We showed that adipose AIF-1 is regulated by changes in body fat mass. AIF-1 gene expression was markedly up-regulated in obese women. These findings support previously published data from genetic studies in humans [16] and mice [17], and give further support to the notion that AIF-1 is an obesity-related gene.

Adipose tissue is composed by a number of different cell types; only around 50% are adipocytes our data suggest that the major source of AIF-1 in human adipose tissue is macrophages, and that the levels of AIF-1 mirror the levels of proinflammatory macrophages in adipose tissue as assessed by the marker ITGAX. We therefore speculate that in WAT, AIF-1 may contribute to the regulation of adipose inflammation. Increases in fat mass prompt accumulation of macrophages in WAT [4,7] which produce a number of chemokines and cytokines [27]. It has been described that AIF-1 stimulates secretion of inflammatory mediators from mouse macrophages [12], indicating that it might be one mediator contributing to WAT inflammation. However, at the present time, the physiological role and mechanism of action of AIF-1 in human WAT remains elusive.

We also observed that AIF-1 expression was associated to a negative metabolic profile (i.e. parameters of insulin resistance). This is in agreement with the finding that adipose AIF-1 is strongly associated with the macrophage marker ITGAX. A low grade of local adipose inflammation has been linked to insulin resistance [32]. We do not know whether AIF-1 is just a marker of adipose inflammation or is directly involved in regulation of adipose insulin sensitivity, maybe by regulating the local production of adipokines in adipose tissue, e.g. by inhibiting production of adiponectin. Interestingly, Fukui M et al., have recently shown that the serum AIF-1 concentrations are positively correlated with an unfavorable metabolic profile with increased fasting plasma glucose and triglycerides, and inversely correlated with high-density lipoprotein cholesterol levels [33]. In a different study, these authors also suggested that AIF-1 could be a marker for diabetic nephropathy as well as activated macrophages [34]. However, we have not investigated whether adipose tissue secreted AIF-1 is involved in systemic inflammation.

Several studies have been published where AIF-1 function was studied by plasmid over-expression in different human cell lines [35,36]. However, this was not physiologically feasible in our experimental settings as we observed that AIF-1 is hardly produced in adipocytes. Moreover, co-culture of macrophages/adipocytes might have been an alternative but was out of the scope of the present study. Another limitation of the present study is that all experiments were carried out in women only. Therefore, evaluation of whether observed effects are also observed in male WAT remains a key issue to be addressed in future. We do not know levels of gender hormones in studied subjects. However, the fact that age had no influence on AIF-1 levels argues against any major influence of menopause on AIF-1. Whether there is an influence of menstrual cycle date on the results presented in this study is very difficult to assess.

## Conclusion

In conclusion, AIF-1 is a novel human adipokine produced mainly by macrophages within WAT that might exert some paracrine effects on fat cells. Adipose AIF-1 is increased in obesity and might participate in the regulation of adipose tissue inflammation and, in turn, insulin resistance.

## Abbreviations

WAT: White adipose tissue; AIF: Allograft-inflammatory factor; BMI: Body mass index; KITT: Constant from insulin tolerance test; SVF: Stroma vascular function.

## Competing interests

The authors declare they have no competing interests.

## Authors’ contributions

SLC, ID, PA participated in the design of the study. PA and ID performed statistical analysis. SLC, PD, ED, AB performed experimental analysis and collecting experimental data. SLC, PA and ID drafted the manuscript. PD, AB and ED made important contributions to manuscript writing. All authors have read and approved the final version of the manuscript.

## Pre-publication history

The pre-publication history for this paper can be accessed here:

http://www.biomedcentral.com/1472-6823/13/54/prepub
